# Surface Dynamic Damage Prediction Model of Horizontal Coal Seam Based on the Idea of Wave Lossless Propagation

**DOI:** 10.3390/ijerph19116862

**Published:** 2022-06-03

**Authors:** Weitao Yan, Junjie Chen, Yi Tan, Rong He, Shaoge Yan

**Affiliations:** 1 School of Surveying and Land Information Engineering, Henan Polytechnic University, Jiaozuo 454003, China; yanweitao@hpu.edu.cn (W.Y.); hero@hpu.edu.cn (R.H.); yanshaoge@hpu.edu.cn (S.Y.); 2 State Collaborative Innovation Center of Coal Work Safety and Clean-Efficiency Utilization, Henan Polytechnic University, Jiaozuo 454003, China

**Keywords:** dynamic prediction model, subsidence velocity, skewed damage distribution, wave lossless propagation, Box–Cox transform algorithm

## Abstract

According to traditional concepts, the movement of overlying strata and surface damage caused by coal mining in horizontal coal seams are symmetrical in terms of spatial distribution. However, in a lot of engineering practices, this symmetry has not been discovered. We often use the symmetry function to establish the profile prediction function of the surface damage, which results in a large difference between the prediction result and the actual situation. To solve this problem, this paper takes subsidence velocity as an example. Firstly, the spatial distribution functions of subsidence velocity on both sides were deduced theoretically. Through comparison, it is found that the change rate of the spatial distribution curve of the coal pillar side subsidence velocity is smoother than that of the goaf side and the subsidence velocity curves are skewed to the left. Secondly, based on the idea of lossless propagation of harmonic waves and idealizing the propagation environment, the spatial propagation relationship of surface subsidence velocity in the time domain is established. Then, the Box–Cox transform function is introduced to improve the normal distribution probability density function, and a new dynamic subsidence prediction model based on the Box–Cox transformation is obtained, which is suitable for the full mining stage. The model is tested by practical cases, the prediction accuracy is better than 7%, and the prediction results can meet the needs of engineering prediction accuracy (10%). The results of this research can enrich the existing subsidence prediction theory and provide theoretical and technical support for the prediction of dynamic surface damage caused by similar mining.

## 1. Introduction

**Figure 1:**
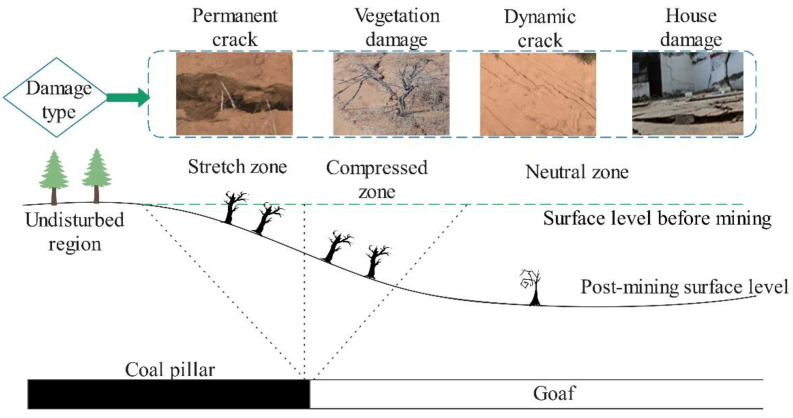
Surface damage type.

Coal resources play a key role in the primary energy structure, especially in China, accounting for 60% of energy produced. However, the large-scale exploitation of coal resources produces a great amount of subsidence. According to relevant statistics, the area of China’s coal subsidence area is about 1.4–2.0 × 10^6^ hm^2^, increasing at the rate of 2.0 × 10^4^ hm^2^ per year [1,2]. Surface subsidence, vegetation damage, house damage, and surface cracking in the subsidence area cause surface damage (Figure 1) [3,4,5].

The surface damage can be divided into dynamic damage and static damage [6,7,8]. The surface damage produced in the process of coal mining is called dynamic damage, while the surface damage produced after mining and surface stability is called static damage. In general, the buried depth of coal seam is large and the dynamic damage of the surface is smaller than the static damage. Therefore, we mainly focus on static damage and conduct a lot of research on static damage. However, with the strategic shift of China’s coal resource exploitation to the west, a large number of high-intensity mining mines have been established in the west, and a large number of coal seam resources with shallow burial, large thickness, small dip angle, and high quality have been rapidly exploited [1]. The dynamic damage of the surface caused by high-intensity coal resource mining is more serious than the static damage [8,9]. In this state, if only the static damage degree is evaluated according to the traditional situation, the damage evaluation result will be seriously distorted. Therefore, it is necessary to study dynamic damage under high-intensity mining conditions.

The surface dynamic damage induced by underground coal mining is a very complex spatiotemporal movement process [10]. To describe the surface dynamic damage phenomenon in the mining area, relevant indexes are usually selected [11]. At present, there are static and dynamic indicators to describe the damage. Static indicators include subsidence, inclination, curvature, horizontal movement, horizontal deformation, distortion, and shear, mainly describing the static damage. The dynamic index is the change rate of the static index in the time domain, that is, the first partial derivative of the static subsidence with time, such as the subsidence velocity, which mainly describes the dynamic damage [12,13,14]. The surface subsidence velocity reflects the severity of damage at a certain surface point at that time. The greater the subsidence velocity, the more intense the dynamic damage of the surface point, and the more serious the damage of ecological elements such as nearby surface soil structures [7,15]. Therefore, the subsidence velocity is often regarded as an effective index to evaluate the degree of surface dynamic damage.

The corresponding relationship between subsidence velocity and surface dynamic damage degree is as follows (Table 1).

To reduce or slow the dynamic damage of mining to the surface, mining damage prevention technology needs to be adopted. However, the premise of effectively adopting mining damage prevention and control technology is to obtain the surface dynamic damage information in advance, that is, to predict the dynamic damage [8,16].

In the aspect of dynamic failure prediction, many scholars have made useful explorations. Since Knothe put forward the Knothe dynamic subsidence time function in 1952 [17], many geometric functions—such as Weibull, Richards, normal distribution time, logistic, Bertalanffy, etc.—have been introduced into the dynamic failure prediction [18,19,20,21,22,23,24,25,26,27,28,29,30,31].

**Figure 2:**
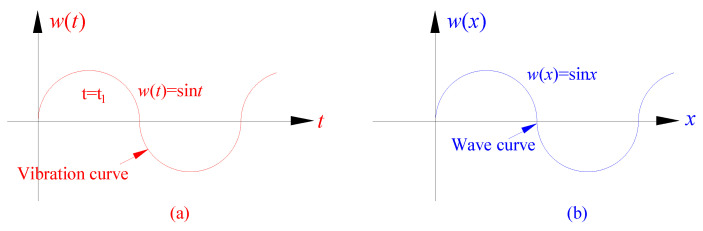
Propagation diagram of wave curve. (**a**) Vibration curve. represents the displacement of a single point at each time. (**b**) Wave curve. represents the displacement of each particle at a certain time.

They mostly start from the time domain, establish a prediction model similar to the vibration curve equation (Figure 2a), and study the deformation development law of a single monitoring point with time, to analyze the dynamic deformation law of a single surface point in the fully mined area in the time domain. This kind of method has achieved good application results in analyzing the deformation law of surface point features (such as lighthouses, high voltage line towers, chimneys, etc.) in coal mining subsidence areas in time domain (Table 2).

However, when analyzing the dynamic deformation of large-scale linear features (such as railways, highways, etc.) and surface features (such as industrial plant areas), the above method is not intuitive and convenient. Therefore, this paper attempts to establish a prediction model similar to the wave curve equation (Figure 2b) and study the dynamic failure from the spatial distribution law of deformation information. Therefore, this paper uses the viscoelasticity theory to carry out the theoretical analysis of dynamic damage, and derives the spatial distribution law of dynamic damage. Then, the Box–Cox function is introduced to improve the probability density function of normal distribution, and the lossless propagation theory of simple harmonics is used for reference, a new prediction model for surface dynamic damage was constructed.

## 2. Theoretical Analysis of Skew Distribution of Surface Dynamic Failure in Mining Area

According to the traditional mining subsidence theory, under the mining conditions of horizontal or near-horizontal coal seams, the distribution of surface dynamics and static damage both have the characteristics of spatial symmetry [7]. However, in the specific engineering practice, it is found that under the mining conditions of horizontal or near-horizontal coal seams, the distribution of surface dynamic and static damage has a certain skewness.

**Figure 3:**
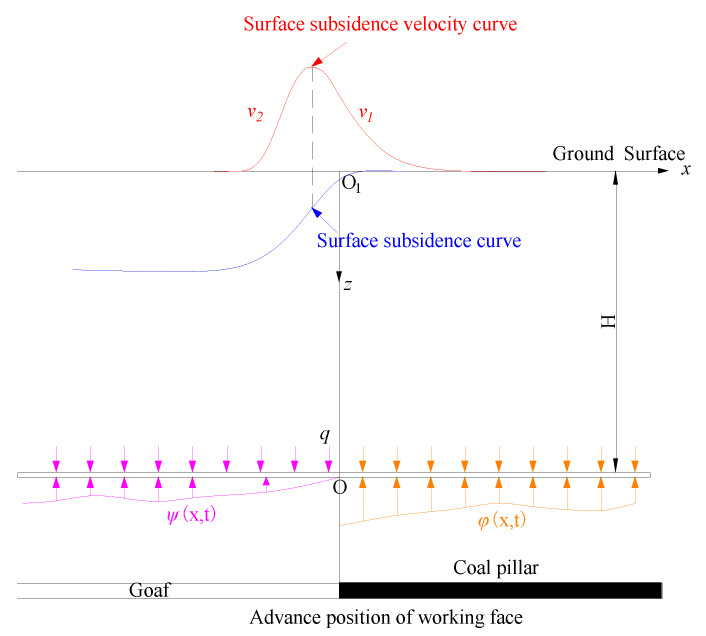
Schematic diagram of skewness distribution of dynamic subsidence.

To analyze the skewness distribution characteristic of dynamic damage, the overburden is regarded as a viscoelastic body. It is assumed that the overburden moves and deforms according to the Kelvin rheological model [10], and the following coordinate system is established (Figure 3).

It is deduced that the subsidence curve model of overburden above the coal pillar and goaf is [10]:

(1) $$\begin{array}{l} W_{1}(x,t)=\left[-\frac{L_{k}-L_{p}}{L_{k}+L_{p}}\sin(\frac{\pi }{L_{p}}x)+\cos(\frac{\pi }{L_{p}}x)\right]*\frac{MP_{z}}{E_{k}}\frac{L_{p}^{2}}{L_{k}^{2}}\left(1-e^{-kt}\right)e^{-\frac{\pi }{L_{p}}x}+\frac{MP_{z}}{E_{p}}x>0\\ W_{2}(x,t)=\left\{e^{\frac{\pi }{L_{k}}x}\left[\frac{L_{k}-L_{p}}{L_{k}+L_{p}}\sin(\frac{\pi }{L_{k}}x)-\cos(\frac{\pi }{L_{k}}x)\right]+1+\frac{L_{p}^{2}}{L_{k}^{2}}\right\}*\frac{MP_{z}}{E_{k}}\left(1-e^{-kt}\right)+\frac{MP_{z}}{E_{p}}x<0 \end{array}$$

where: $W_{1}(x,t)$ and $W_{2}(x,t)$ are the subsidence curve equations of overburden above the coal pillar side and goaf side, respectively. $L_{P}$ and $L_{k}$ are the half wavelength of overburden pressure wave on the side of coal pillar and goaf, respectively. M is the mining thickness; $P_{z}$ is the initial stress, $P_{z}=\gamma H$; $E_{k}$ and $E_{P}$ are the elastic modulus of the a side and coal pillar side, respectively. η is the coefficient of viscosity k=E/η.

Calculate the partial derivative of Formula (1) in the time domain to obtain the subsidence velocity curve of overburden above the coal pillar side and goaf side:

(2) $$\begin{array}{ll} v_{1}(x,t) & =\frac{\partial W_{1}(x,t)}{\partial t}\\ & =ke^{-kt}e^{-\frac{\pi }{L_{p}}x}\frac{MP_{z}}{E_{k}}\frac{L_{p}^{2}}{L_{k}^{2}}*\left[-\frac{L_{k}-L_{p}}{L_{k}+L_{p}}\sin(\frac{\pi }{L_{p}}x)+\cos(\frac{\pi }{L_{p}}x)\right]\;x>0\\ v_{2}(x,t) & =\frac{\partial W_{2}(x,t)}{\partial t}\\ & =\left\{e^{\frac{\pi }{L_{k}}x}\left[\frac{L_{k}-L_{p}}{L_{k}+L_{p}}\sin(\frac{\pi }{L_{k}}x)-\cos(\frac{\pi }{L_{k}}x)\right]+1+\frac{L_{p}^{2}}{L_{k}^{2}}\right\}*ke^{-kt}\frac{MP_{z}}{E_{k}}\;x<0 \end{array}$$

where: $v_{1}(x,t)$ and $v_{2}(x,t)$ are the subsidence velocity curve equations of overburden above the coal pillar side and goaf side, respectively.

Then, calculate the partial derivative of the subsidence velocity curve Equation (2) in the axial direction to obtain the change rate of the subsidence velocity curve on both sides of the advancing position of the working face:

(3) $$\begin{array}{cc} \frac{\partial v_{1}\left(x,t\right)}{\partial x}= & \frac{\partial^{2}W_{1}\left(x,t\right)}{\partial t\partial x}\\ = & -\frac{\pi }{L_{p}}ke^{-kt}e^{-\frac{\pi }{L_{p}}x}\frac{MP_{z}}{E_{k}}\frac{L_{p}^{2}}{L_{k}^{2}}*\left[\cos\left(\frac{\pi }{L_{p}}x\right)-\frac{L_{k}-L_{p}}{L_{k}+L_{p}}\sin\left(\frac{\pi }{L_{p}}x\right)\right]\\ \; & +ke^{-kt}e^{-\frac{\pi }{L_{p}}x}\frac{MP_{z}}{E_{k}}\frac{L_{p}^{2}}{L_{k}^{2}}*\left[-\frac{L_{k}-L_{p}}{L_{k}+L_{p}}\cos\left(\frac{\pi }{L_{p}}x\right)\frac{\pi }{L_{p}}-\frac{\pi }{L_{p}}\sin\left(\frac{\pi }{L_{p}}x\right)\right]\\ \frac{\partial v_{2}\left(x,t\right)}{\partial x}= & \frac{\partial^{2}W_{2}\left(x,t\right)}{\partial t\partial x}\\ = & ke^{-kt}\frac{MP_{z}}{E_{k}}\frac{\pi }{L_{k}}e^{\frac{\pi }{L_{k}}x}*\left[\frac{L_{k}-L_{p}}{L_{k}+L_{p}}\sin\left(\frac{\pi }{L_{k}}x\right)-\cos\left(\frac{\pi }{L_{k}}x\right)\right]\\ \; & +ke^{-kt}\frac{MP_{z}}{E_{k}}\frac{\pi }{L_{k}}e^{\frac{\pi }{L_{p}}x}*\left[\frac{L_{k}-L_{p}}{L_{k}+L_{p}}\cos\left(\frac{\pi }{L_{k}}x\right)+\sin\left(\frac{\pi }{L_{k}}x\right)\right] \end{array}$$

where $\frac{\partial v_{1}\left(x,t\right)}{\partial x}$ and $\frac{\partial v_{2}\left(x,t\right)}{\partial x}$ are the spatial change rates of the subsidence velocity curve of the overburden above the coal pillar side and the goaf side, respectively, and their absolute values can reflect the steepness of the subsidence velocity curve on both sides.

Compared with the coal pillar, the strength of the caving rock block in goaf is small, so the following relationship can be obtained:

(4) $$\left\{\begin{array}{l} E_{k}<E_{p}\\ L_{p}<L_{k} \end{array}\right.$$

By bringing Formula (4) into (3), we can obtain

(5) $$\left|\frac{\partial v_{1}(x,t)}{\partial x}\right|<\left|\frac{\partial v_{2}(x,t)}{\partial x}\right|$$

According to Formula (5), the change rate of surface subsidence velocity along the *x*-axis on the coal pillar side is less than that on the goaf side. That is, the subsidence velocity curve on the coal pillar side is slow, the subsidence velocity curve on the goaf side is steep, and the subsidence velocity curves on both sides are not symmetrically distributed at the maximum subsidence velocity point. When the movement and deformation of overburden is transmitted to the surface, it will also show the same law on the surface, that is, the surface subsidence velocity curve is skewed in space, and the surface subsidence velocity curve on the coal pillar side is gentler than that on the goaf side.

To sum up, under the mining conditions of horizontal coal seam or near horizontal coal seam, the surface dynamic subsidence curve (spatial distribution curve of subsidence velocity) presents the spatial skewness along the section of the advancing direction of the working face. Therefore, to improve the prediction accuracy of dynamic failure, it is necessary to establish a dynamic failure prediction model in line with the law of skew distribution.

## 3. Establishment of Dynamic Failure Prediction Model of Surface

Based on the above analysis, the idea of establishing the prediction model is mainly divided into the following three steps:

(1) Simplifying the subsidence environment.
(2) Based on the lossless propagation theory of plane simple harmonic wave, the space-time propagation mode of damage is established.
(3) Box–Cox function is introduced to improve the probability density function of normal distribution, and a dynamic failure prediction model suitable for skew distribution is given.

The details are as follows.

### 3.1. Model Assumptions

The realistic scene of subsidence is often complicated, which needs to be idealized and simplified when establishing the prediction model. Before establishing the model, the following assumptions should be made:

(1) Horizontal layered deposition of overburden and coal seam;
(2) The surface is relatively flat without large fluctuation;
(3) The working face is rectangular;
(4) There are no major geological structures and geological events in the study area;
(5) The study period is in the full mining stage, and the study area is located in the full mining area.

### 3.2. Spatial Propagation Mode of Subsidence Velocity Curve

**Figure 4:**
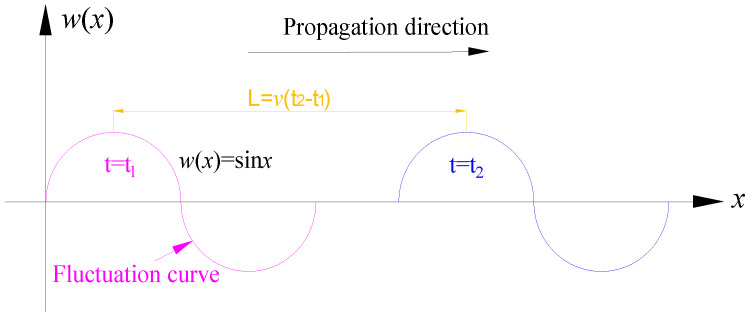
Propagation diagram of wave curve.

According to the wave theory, the wave curve is the spatial distribution curve of the amplitude of each particle at a certain time. In the process of lossless propagation of a plane simple harmonic wave, the whole waveform remains unchanged and propagates forward along the propagation direction at the wave velocity *v* (Figure 4).

It is assumed that the propagation of the surface subsidence velocity curve is also a non-destructive propagation, that is, in the full mining stage, the waveform of the subsidence velocity curve remains unchanged in the propagation process and propagates forward along the advancing direction of the working face at the mining speed. With the advance of the working face, the surface subsidence velocity curve at t_2_ can be considered obtained after the subsidence velocity curve at t_1_ translates the distance L along the propagation direction.

Based on the above idea, after the prediction function model of surface subsidence velocity at a certain time is obtained, the rest time can be obtained by translating the prediction model for a certain distance along the advancing direction of the working face. There are three calculation methods for the translation:

① Multiplying the mining speed by the interval time;

② Calculating the distance between the maximum subsidence velocity points in two time periods;

③ Calculating the advancing distance of the working face in two time periods.

Because the underground mining speed is not uniform, the calculation method ① is inconvenient, so method ② or method ③ is usually used in application. The calculation process of method ② is as follows:

The calculation formula for the position of the maximum subsidence velocity point is

(6) $$x_{v_{\max}}=L-H\cot\varphi$$

where *L* is the advancing distance of the working face; *H* is the mining depth; and φ is the lag angle of the maximum subsidence velocity.

The distance between the maximum subsidence velocity points at two times is calculated by the following formula.

(7) $$d=x_{v_{\max-1}}-x_{v_{\max-2}}=D_{1}-D_{2}$$

According to Equation (7), the translation distance between the maximum subsidence velocity curves at two times in the full mining stage is the advancing distance of the working face in this period.

### 3.3. Establishment of Prediction Model

Box–Cox transformation is a parameterized generalized power data transformation method in mathematical modeling, which aims to stabilize the variance, reduce the non-normality of data, and enhance the effectiveness of correlation measurement [32], which can bring the data closer to the normal distribution. The transformation formula is

(8) $$y(\lambda )=\left\{\begin{array}{l} \frac{y^{\lambda }-1}{\lambda },\;\lambda \neq 0\\ \ln y,\;\lambda =0 \end{array}\right.$$

where $y\left(\lambda \right)$ is the new variable obtained by transformation, and λ is the transformation parameter.

Through the above transformation of the dependent variable observation $\left\{y_{1},y_{2},y_{3},\ldots ,y_{n}\right\}$, the transformed vector $y^{\left(\lambda \right)}=\left\{y_{1}^{\lambda },y_{2}^{\lambda },y_{3}^{\lambda },\ldots ,y_{n}^{\lambda }\right\}$ basically conforms to the normal distribution.

The distance from the stope line is regarded as the sample, and the subsidence velocity is regarded as the frequency. Firstly, the distance observation value is dimensionless

(9) $${x^{\prime }}_{i}=\frac{x_{i}}{\bar{x}}$$

where $\bar{x}$ is the average distance. After dimensionless data, new vector data are obtained by using Box–Cox transformation formula.

(10) $$y_{i}=\frac{{x^{\prime }}_{i}{}^{\lambda }-1}{\lambda }$$

(11) $$y_{i}=\frac{x_{i}{}^{\lambda }-{\bar{x_{}}}^{\lambda }}{\lambda {\bar{x_{}}}^{\lambda }}$$

(12) $$y_{i}\sim N(0,\sigma^{2})$$

Therefore, when *x* > 0,

(13) $$F(y)=P(Y\leq y)=P(Y\leq \frac{x_{i}{}^{\lambda }-{\bar{x_{}}}^{\lambda }}{\lambda {\bar{x_{}}}^{\lambda }})=\phi (\frac{x_{i}{}^{\lambda }-{\bar{x_{}}}^{\lambda }}{\lambda {\bar{x_{}}}^{\lambda }})$$

where ϕ represents the distribution function of normal distribution $N\left(0,\sigma^{2}\right)$. The transformed data conforms to the normal distribution, and the density function of the original variable can be solved by means of the normal distribution probability density function.

When *x* > 0, the probability density function of *x* is

(14) $$f_{x}(x)=\phi^{\prime }(\frac{x_{i}{}^{\lambda }-{\bar{x_{}}}^{\lambda }}{\lambda {\bar{x_{}}}^{\lambda }})=\varphi^{\prime }(\frac{x_{i}{}^{\lambda }-{\bar{x_{}}}^{\lambda }}{\lambda {\bar{x_{}}}^{\lambda }})=\frac{x_{i}{}^{\lambda -1}}{\sqrt{2\pi }\sigma {\bar{x_{}}}^{\lambda }}\exp[-\frac{{\left(x_{i}{}^{\lambda }-{\bar{x_{}}}^{\lambda }\right)}^{2}}{2\sigma^{2}\lambda^{2}{\bar{x_{}}}^{2\lambda }}]$$

When x≤0, the probability density function of *x* is

(15) $$f_{x}\left(x\right)=0$$

where σ is the standard deviation after Box–Cox transformation; φ is the probability density function of normal distribution $N\left(0,\sigma^{2}\right)$; and λ is a variation parameter.

Based on the measured subsidence velocity under the condition of full mining at a certain time, the prediction curve formula of subsidence velocity at that time is obtained

(16) $$v(x)=\frac{a}{\sqrt{2\pi }bc^{\lambda }}\exp[-\frac{{\left(x^{\lambda }-c^{\lambda }\right)}^{2}}{2b^{2}\lambda^{2}c^{2\lambda }}]x^{\lambda -1}$$

where $v\left(x\right)$ is the subsidence velocity, mm/d; x is the distance from stoping line, m; and a,b,c,λ is the model parameter.

Since this time, the prediction model of surface subsidence velocity when the working face advances forward by a distance *d* is

(17) $$v(x)=\frac{a}{\sqrt{2\pi }bc^{\lambda }}\exp[-\frac{{\left({\left(x+d\right)}^{\lambda }-c^{\lambda }\right)}^{2}}{2b^{2}\lambda^{2}c^{2\lambda }}]{\left(x+d\right)}^{\lambda -1}$$

### 3.4. Model Parameters and Mathematical Characteristics

According to the control effect of various parameters in the model on the curve shape or quantity value [32], the parameters a and b are quantity value parameters, c and λ are the shape parameters.

**Figure 5:**
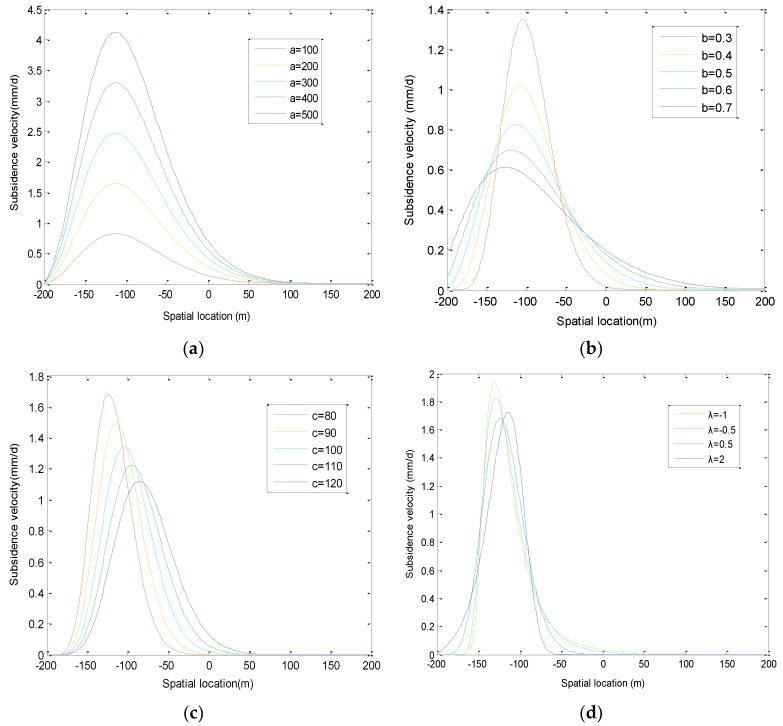
Model parameter analysis. (**a**) Influence of parameter a on curve shape, (**b**) Influence of parameter b on curve shape, (**c**) Influence of parameter c on curve shape, (**d**) Influence of parameter λ on curve shape.

Parameters a and b are closely related to the severity of surface damage. The more intense the surface damage is, the larger the magnitude parameter a is, and the smaller the magnitude parameter b is (Figure 5a,b).

Parameter c is the shape parameter, and it plays a decisive role in curve shape. c represents the degree of skewness of the curve (Figure 5c,d). With the increase in the c value, the curve skewness decreases gradually.

λ is the transformation parameter, and it can be determined by the maximum likelihood method. λ reflects the skewness degree between the data and the normal distribution, when λ < 0, the original data is left biased; when λ > 0, the original data is biased to the right. λ = 0 indicates that the original data is normally distributed.

### 3.5. Application Scope of Prediction Model

This prediction model is a statistical mathematical model and it fits in with the skewed curve characteristics. Therefore, the application is limited. When the movement of strata above the gob is only affected by self-weight and overburden pressure, the prediction precision is high. However, when a place has a wide range of geological structures (e.g., faults, folds) or geological events (e.g., earthquake), this prediction model will be not suitable and the prediction precision is low.

## 4. Example Verification

### 4.1. Example Verification of the Spatial Distribution Law of Surface Skewed Destruction

Working face 2407 is selected for analysis. It belongs to Halagou coal mine. Halagou coal mine is located in the contiguous of Inner Mongolia and Shaanxi Province and in the border area of the Loess Plateau and the Mu Us desert. There are rich coal resources and China’s first 100 million tons of coal production base. The features of geo-mining conditions in this area are shallow buried depth, thin bedrock, thick loose layer, large mining height, and fast mining velocity.

**Figure 6:**
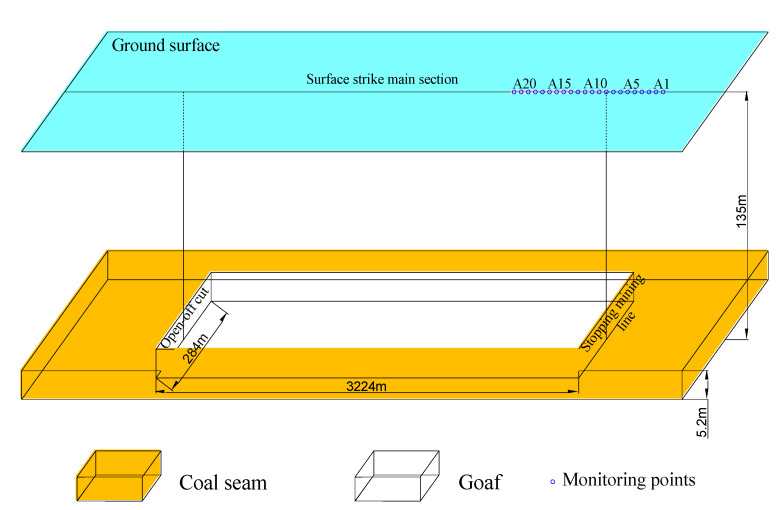
Overview of working face and layout of observation station.

Working face 2407 has a mining depth of 135 m, of which the bedrock is 73 m thick, the loose layer is 57 m thick, and the coal thickness is 5.2 m. The size of the working face is 3224 × 284 m, the inclination angle of the coal seam is about 1°, and the advancing speed is 15 m/d [8]. A surface mobile observation station is arranged at one end of the stop mining line along the strive direction of the working face (Figure 6).

**Figure 7:**
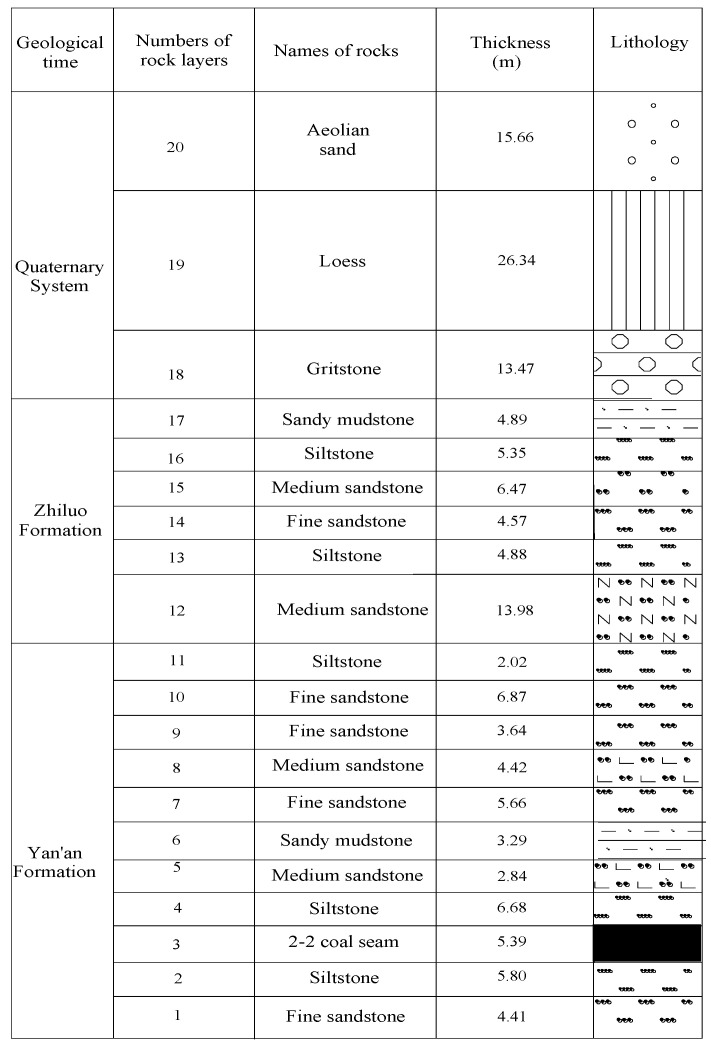
The borehole log of working face 2407.

According to the geo-mining conditions and borehole histogram of working face 2407 (Figure 7), the lithology of the overlying strata in this working face is evaluated as medium-hard.

**Figure 8:**
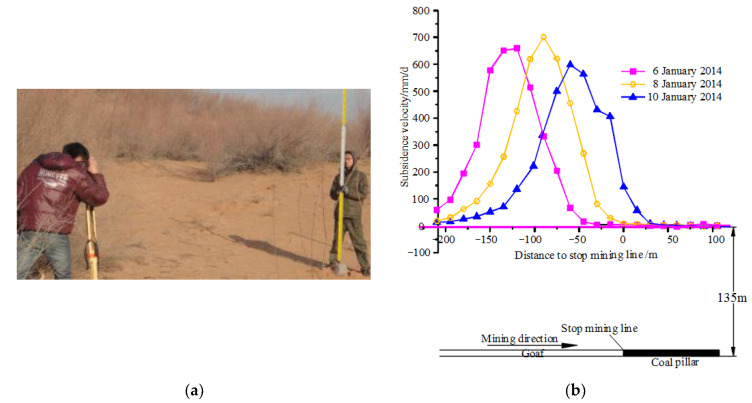
Monitoring method and data [8]. (**a**) Monitoring method, (**b**) monitoring data.

After the surface monitoring points are arranged, the leveling method is used to measure the elevation of all monitoring points (Figure 8a). The surface subsidence value can be calculated by the following formula.

(18) $$w_{n,i}=H_{n0}-H_{ni}$$

where $w_{n,i}$ is the subsidence value of point *n* measured at *i* times; $H_{n0}$ and $H_{ni}$ are the elevation value of point *n* measured at No. *i* and first time, mm.

The surface subsidence velocity can be calculated by the following formula.

(19) $$v_{n}=\frac{w_{n,i+1}-w_{n,i}}{t}$$

where $v_{n}$ is the subsidence velocity of point *n*; $w_{n,i+1}$ and $w_{n,i}$ are the subsidence value of point *n* measured at No. *i* + 1 and No. *i* times, mm; t is the interval between two measurements, *d*.

The surface subsidence velocity data on 6, 8, and 10 January 2014 were selected for analysis. There are 22 monitoring points with known data, and their monitoring data are shown in Figure 8b.

The normal distribution and skewed distribution can be described by the skewness coefficient, which reflects the degree to which the data distribution deviates from the center position. Under normal distribution conditions, its skewness coefficient is equal to 0. When the skewness coefficient > 0, it is right skewed, and when the skewness coefficient < 0, it is left skewed. The formula for calculating the skewness coefficient is

(20) $$SK=\frac{\bar{X}-M_{0}}{\sigma }$$

where *SK* is the skewness coefficient, *M*_0_ is the mode, and *σ* is the standard deviation.

Calculate its skewness coefficient to obtain Table 3:

From Table 3, it can be concluded that the skewness coefficient of the on-site surface subsidence velocity data on the 6th, 8th, and 10th are smaller than 0; that is, the three on-site surface subsidence velocity data are all skewed to the left, and the subsidence velocity curve on the coal pillar side is relatively flat. The gob side is steeper, which verifies the theoretical analysis results of Section 2.

### 4.2. Instance Accuracy Verification of Predictive Models

Based on the measured data of the surface subsidence velocity of the mining area on 6 January 2014, Formula (16) is used to perform curve fitting with the least square as the criterion, nonlinear fitting was performed to obtain the estimated parameters

(21) a=54987.418,b=0.254,c=128.093,λ=0.976

The expected equation is obtained

(22) $$v(x)=\frac{54987.42}{\sqrt{2\pi }\times 0.254\times 128.093^{0.976}}\times \exp[-\frac{{\left(x^{0.976}-128.093^{0.976}\right)}^{2}}{2\times 0.254^{2}\times 0.976^{2}\times 128.093^{1.952}}]\times x^{-0.024}$$

Then, the prediction function model is

(23) $$v(x)=\frac{54987.42}{\sqrt{2\pi }\times 0.254\times 128.093^{0.976}}\times \exp[-\frac{{\left({\left(x+d\right)}^{0.976}-128.093^{0.976}\right)}^{2}}{2\times 0.254^{2}\times 0.976^{2}\times 128.093^{1.952}}]\times {\left(x+d\right)}^{-0.024}$$

**Figure 9:**
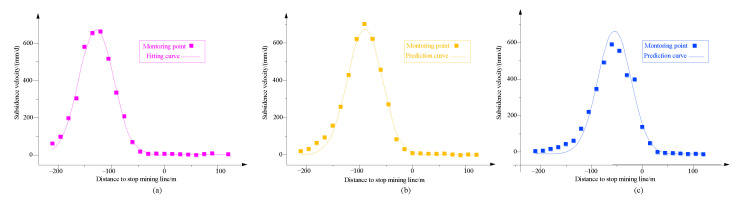
Fitting curve and prediction curve. (**a**) 6 January 2014, (**b**) 8 January 2014, (**c**) 10 January 2014.

Using the prediction parameters obtained by fitting the data on the 6th (Figure 9a), the surface subsidence velocity on the 8th and 10th were predicted. The predicted results are shown in Figure 9b,c.

### 4.3. Prediction Accuracy Analysis

The prediction accuracy is measured by two indicators: medium error and relative medium error. The formula for calculating the medium error is

(24) $$m=\sqrt{\frac{\left[\Delta \Delta \right]}{n}}$$

The formula for calculating the relative medium error is

(25) $$f=\frac{\left|m\right|}{V_{\max}}$$

where Δ is the difference between the predicted and the actual measurement, mm; [] is the summation; *n* is the number of predicted points; and $V_{\max}$ is the maximum value of the subsidence velocity, mm.

After analyzing and testing the prediction model, the accuracy analysis results of the 8th and 10th days can be obtained. The results are shown in Table 4.

From the above analysis results, it can be seen that the relative median error between the measured results of the prediction model and the corresponding points of the fitting curve is less than 7%. Generally speaking, the requirement of the engineering application for prediction accuracy is that the relative median error is less than 10%. That is to say, this prediction model meets the prediction accuracy requirements of the project.

## 5. Conclusions

(1) Under the condition of horizontal or near-horizontal coal seam mining, the subsidence velocity curve of the overburden and the surface on both sides of the working face advancing position is not spatially symmetric with respect to the maximum subsidence velocity position. The subsidence velocity curve on the coal pillar side is gentler than that on the gob side, showing a right skew state.
(2) Based on the lossless propagation theory of simple harmonic wave, the spatiotemporal propagation mode of the subsidence velocity curve in the fully mining stage is constructed.
(3) The Box–Cox transformation function is introduced to improve the probability density function of normal distribution, and a new model for predicting the ground surface skewed damage in the full mining stage is proposed.
(4) Combined with an example, the right skewness spatial distribution law of dynamic damage and the prediction accuracy of the dynamic prediction model are tested. The measured skewness law verifies the above theoretical analysis results. The prediction model has high accuracy, the relative error is less than 7%, which can meet the needs of engineering.

## Figures and Tables

**Table 1:** Surface damage level and corresponding maximum allowable subsidence velocity values.

Surface Damage Level	0	1	2	3	4
Allowable value of maximum subsidence velocity	1 mm/d	3 mm/d	6 mm/d	12 mm/d	18 mm/d

**Table 2:** Differences with traditional analysis methods.

	Traditional Method (Time Domain Analysis)	Method in This Paper (Spatial Domain Analysis)
Representative function	Weibull, Richards, Normal distribution time, Logistic, Bertalanffy	Box–Cox
Similar wave curve types	vibration curve	wave curve
Analysis ideas	analyze the dynamic deformation law of a single surface point in the time domain	study the dynamic failure from the spatial distribution law of deformation information
Research object	a single surface point	multiple surface monitoring points

**Table 3:** Skewness analysis.

Date	Skewness Coefficient
6 January 2014	−2.171
8 January 2014	−2.294
10 January 2014	−2.140

**Table 4:** Accuracy analysis of model prediction.

	8 January 2014	10 January 2014
Medium error/mm	20.28	39.64
Relative medium error	2.9%	6.6%

## Data Availability

Not applicable.
